# Rewiring yeast acetate metabolism through *MPC1* loss of function leads to mitochondrial damage and decreases chronological lifespan

**DOI:** 10.15698/mic2014.12.178

**Published:** 2014-11-18

**Authors:** Ivan Orlandi, Damiano Pellegrino Coppola, Marina Vai

**Affiliations:** 1 SYSBIO Centre for Systems Biology Milano, Italy.; 2 Dipartimento di Biotecnologie e Bioscienze, Università di Milano-Bicocca, Piazza della Scienza 2, 20126 Milano, Italy.

**Keywords:** acetyl-CoA, chronological aging, Mcp1, mitochondria, pyruvate, Saccharomyces cerevisiae

## Abstract

During growth on fermentable substrates, such as glucose, pyruvate, which is the end-product of glycolysis, can be used to generate acetyl-CoA in the cytosol via acetaldehyde and acetate, or in mitochondria by direct oxidative decarboxylation. In the latter case, the mitochondrial pyruvate carrier (MPC) is responsible for pyruvate transport into mitochondrial matrix space. During chronological aging, yeast cells which lack the major structural subunit Mpc1 display a reduced lifespan accompanied by an age-dependent loss of autophagy. Here, we show that the impairment of pyruvate import into mitochondria linked to Mpc1 loss is compensated by a flux redirection of TCA cycle intermediates through the malic enzyme-dependent alternative route. In such a way, the TCA cycle operates in a “branched” fashion to generate pyruvate and is depleted of intermediates. Mutant cells cope with this depletion by increasing the activity of glyoxylate cycle and of the pathway which provides the nucleocytosolic acetyl-CoA. Moreover, cellular respiration decreases and ROS accumulate in the mitochondria which, in turn, undergo severe damage. These acquired traits in concert with the reduced autophagy restrict cell survival of the mpc1∆ mutant during chronological aging. Conversely, the activation of the carnitine shuttle by supplying acetyl-CoA to the mitochondria is sufficient to abrogate the short-lived phenotype of the mutant.

## INTRODUCTION

Aging of postmitotic quiescent mammalian cells has been modelled in the yeast* Saccharomyces cerevisiae* by its chronological lifespan (CLS) 1,2. CLS represents the length of time a culture of nondividing cells remains viable in stationary phase: viability is assessed by the ability to resume growth upon return to rich medium 3. Evidence to date indicates that chronological aging is intimately regulated by signaling pathways which sense nutrient availability, namely TORC1-Sch9 and Ras-PKA, and carbon metabolism 4,5. In this context, emerging data on some metabolites and nutrient manipulation/dietary regimens which proved to modulate aging not only in yeast but also in evolutionary diverse organisms have opened up new opportunities for therapeutic interventions promoting healthy aging in humans 6,7. In particular, among the main metabolic intermediates, acetyl-CoA is increasingly being acknowledged as an important regulator of longevity 8,9,10. This metabolite is the activated form of acetate obtained via a thioester linkage with coenzyme A which cells use for macromolecule biosynthesis. Furthermore, in the mitochondria it is a crucial substrate for energy production since it fuels the TCA cycle and consequently the production of reducing equivalents which enter the electron transport chain and support the oxidative phosphorylation. In addition, acetyl-CoA also supplies the acetyl group for protein acetylation, a dynamic posttranslational modification which occurs on a wide range of substrates, including histones and many metabolic enzymes, thus connecting metabolism, epigenetics and transcriptional regulation 11,12,13,14. Recently, by manipulating the major routes of acetyl-CoA formation in yeast and mammalian cells, it has been shown that the nucleocytosolic pool of acetyl-CoA acts as a dominant suppressor of cytoprotective autophagy during aging 8,10. In line with this, during aging, histone hypoacetylation correlates with enhanced expression of *ATG* genes and induction of autophagy 15. This is a degradative process which is crucial for the maintenance of cellular homeostasis by removing misfolded/damaged or “obsolete” proteins and organelles, including mitochondria. It becomes fundamental in nondividing cells where the intracellular damage cannot be “diluted” 16. Nutrient depletion and inactivations of genes in the central nutrient signaling pathways are known inducers of autophagy 17.

**Figure 1:**
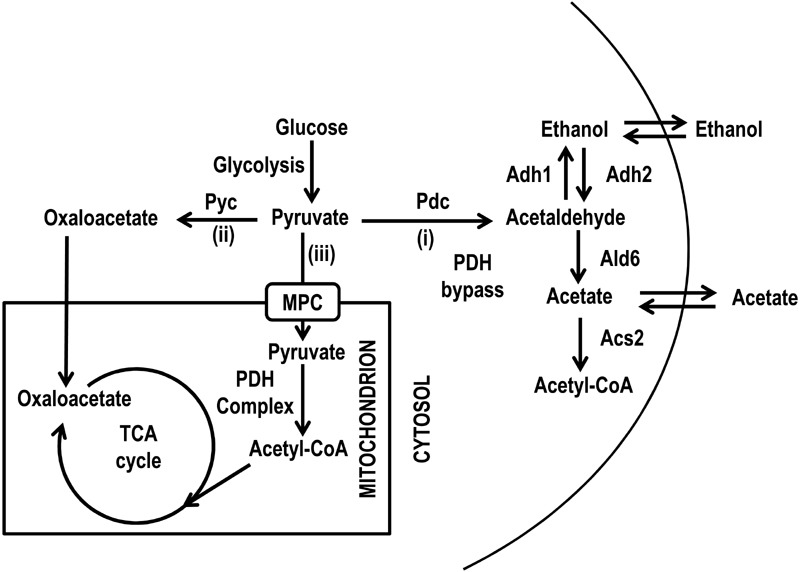
FIGURE 1: Scheme of metabolic pathways allowing pyruvate utilization. The three pathways which originate from pyruvate after (i) decarboxylation to acetaldehyde, (ii) carboxylation to oxaloacetate and (iii) oxidative decarboxylation to acetyl-CoA are schematically shown. Acs, acetyl-CoA synthase; Adh, alcohol dehydrogenase; Ald, aldehyde dehydrogenase; MPC, mitochondrial pyruvate carrier; Pdc, pyruvate decarboxylase; PDH, pyruvate dehydrogenase; Pyc, pyruvate carboxylase.

In *S.cerevisiae*, the nucleocytosolic pool of acetyl-CoA is synthesized by the acetyl-CoA synthetase 2 (Acs2) by activation of acetate in an ATP-dependent reaction. This enzyme is known as the glycolytic isoform 18 and besides its role in carbon metabolism it is required for histone acetylation 19. The mitochondrial acetyl-CoA pool is generated by the Acs1 (the gluconeogenic isoform) and by the acetyl-CoA hydrolase 1 (Ach1) which catalyzes the transfer of the CoASH moiety from succinyl-CoA to acetate 20. Moreover, according to culture conditions, acetyl-CoA can be formed and utilized in different ways. During growth on fermentable substrates, such as glucose, it is generated from pyruvate. This compound is the end-product of glycolysis and is a key node in the branching point between respiratory metabolism and alcoholic fermentation as well as assimilatory and dissimilatory metabolic reactions 21. At the branching point, it can follow three major fates (Fig. 1): (i) decarboxylation to acetaldehyde which generates acetyl-CoA by the pyruvate dehydrogenase (PDH) bypass; (ii) anaplerotic carboxylation to oxaloacetate and (iii) the direct oxidative decarboxylation to acetyl-CoA by the PDH complex, which is located in the mitochondrial matrix. Pyruvate can cross the outer mitochondrial membrane while the passage across the inner mitochondrial membrane requires the mitochondrial pyruvate carrier (MPC) 22,23. This carrier effectively represents a link between cytosolic pyruvate metabolism and the TCA cycle. Loss of the major structural subunit Mpc1 results in defective mitochondrial pyruvate uptake 22 and, during chronological aging, in a short-lived phenotype accompanied by an age-dependent loss of autophagy 8.

In this work we investigated the metabolic changes underlying *MPC1* loss of function. We found that *mpc1*∆ cells make up for their impairment in mitochondrial pyruvate with a metabolic rewiring which involves several intermediates of the mitochondrially localized TCA cycle and the cytosolic glyoxylate shunt but ultimately results in a pro-aging process.

## RESULTS AND DISCUSSION

### Lack of Mpc1 is accompanied by an increase of Ald enzymatic activities

**Figure 2:**
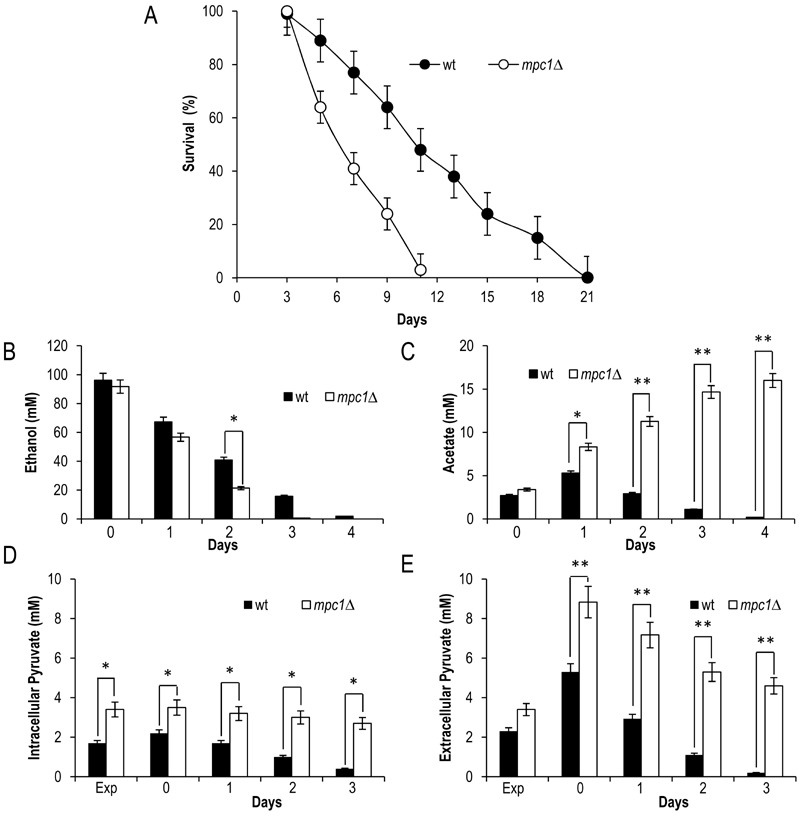
FIGURE 2:*MPC1* inactivation shortens CLS in concert with increased extracellular acetate and pyruvate. Wild type (wt) and *mpc1*Δ mutant cells were grown in minimal medium/2% glucose and the required supplements in excess (see Materials and Methods) and followed up to stationary phase. **(A)** CLS of wt and *mpc1*Δ mutant cells. At each time-point, survival was determined by colony-forming capacity. 72 h after the diauxic shift (Day 3) was considered the first age-point (see Materials and Methods). Day 0, diauxic shift. Data refer to mean values of three independent experiments. Standard deviations (SD) are indicated. Bar charts of extracellular ethanol **(B)** and acetate **(C)** concentrations at different time points after the diauxic shift (Day 0). In parallel, intracellular **(D)** and extracellular **(E)** pyruvate concentrations were measured. Exp, exponential growth phase. Data refer to mean values of three independent experiments. SD is indicated. Statistical significance as assessed by one-way ANOVA test is indicated (* P ≤ 0.05 and ** P ≤ 0.01).

Since an impairment in the import of pyruvate into mitochondria linked to *MPC1* deletion significantly restricted CLS (Fig. 2A) 8, we decided to analyze in more detail the metabolic changes underlying this short-lived phenotype. Initially, in the context of a standard CLS experiment 3, we measured the levels of some metabolites such as pyruvate, ethanol and acetate. These last two compounds are produced during glucose fermentation following decarboxylation of cytosolic pyruvate to acetaldehyde by pyruvate decarboxylase (Pdc) (Fig. 1). Only upon glucose depletion, does the diauxic shift occurs and yeast cells switch to a respiration-based metabolism of the fermentation C2 by-products. Finally, when these carbon/energy sources are fully exhausted, cells enter a quiescent stationary phase. At the diauxic shift, in the *mpc1*∆ culture the amount of ethanol and acetate was similar to that in the wild type (wt) culture (Fig. S1, 2B and C). Differently, during the post-diauxic phase, in the mutant the consumption of ethanol, which is re-introduced into the metabolism via its oxidation to acetate (Fig. 1), was not affected significantly compared to that in the wt (specific consumption rate, qEtOH, of 1.43 ± 0.04 mmol•g•DW^-1^•h^-1 ^for the mutant and 1.12 ± 0.06 mmol•g•DW^-1^•h^-1 ^for the wt) (Fig. S1 and 2B), while the acetate continued to accumulate in the medium (Fig. 2C). Such a prolonged secretion of acetate throughout the ethanol consumption phase suggests that in the *mpc1*∆ mutant there is an imbalance between acetate production rate from acetaldehyde and its conversion rate into acetyl-CoA. In fact, the acetate transport relies on an active transport for the dissociated form of the acid (subjected to glucose repression) accompanied by passive/facilitated diffusion of the undissociated acid 24. During the post-diauxic phase the pH of the medium is far below the pKa of acetic acid (4.75) 25 and according to the Henderson-Hasselbalch equation, acetic acid is substantially undissociated: 98.6 % at pH 2.9 (the value we measured at Day 3 after the diauxic shift). Consequently, in a condition where transmembrane diffusion strongly prevails over the active transport, the acetate export/import will take place according to the gradient between the intracellular and extracellular concentrations of acetate.

Concerning intracellular pyruvate, its concentration was higher in the *mpc1*∆ mutant compared to that in the wt not only in exponential phase, as already observed by 22, but also at/after the diauxic shift (Fig. 2D). This was also associated with an increase in the extracellular pyruvate (Fig. 2E) which reflects an overflow of pyruvate within the cytosol. Similar results were obtained when the experiments were also performed by growing the histidine-prototroph *mpc1*∆ mutant (*mcp1*∆::*HIS3*) in a histidine-supplemented medium as previously carried out for the wt (Fig. S2) indicating that the different composition of amino acids in the medium does not affect the results.

Afterwards, we measured the enzymatic activities of alcohol dehydrogenases (Adhs) catalysing the interconversion of acetaldehyde and ethanol 26 and of acetaldehyde dehydrogenases (Alds) which produce acetate by oxidizing the acetaldehyde generated from pyruvate during fermentation and that obtained during ethanol oxidation (Fig. 1).

**Figure 3:**
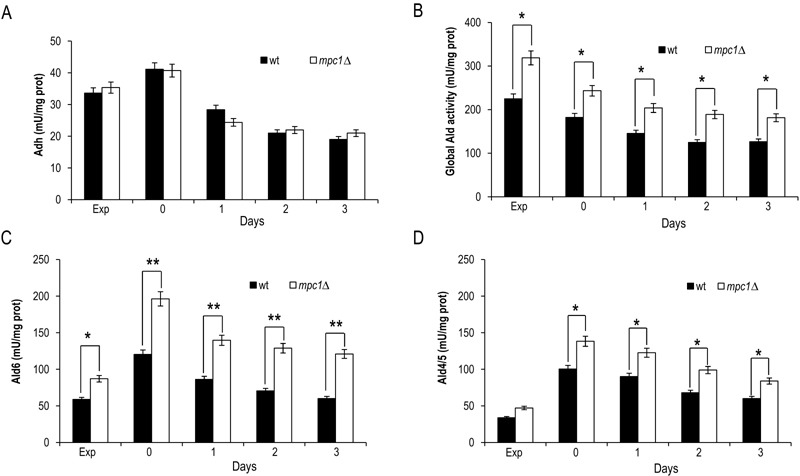
FIGURE 3:In *mpc1*∆ cells the extracellular abundance of acetate correlates with enhanced Ald enzymatic activity. Bar charts of total Adh **(A),** total Ald **(B**), Ald6 **(C)** and Ald4/5 **(D)** enzymatic activities measured at the indicated time points for wt and *mpc1Δ* mutant cells grown as in Figure 2. Exp, exponential growth phase. Day 0, diauxic shift. Data refer to mean values determined in three independent experiments. SD is indicated. * P ≤ 0.05 and ** P ≤ 0.01.

No significant difference was found between the wt and the *mpc1*∆ strain in the Adh activity levels in exponential phase (Fig. 3A), where the Adh1 isoenzyme is chiefly responsible for ethanol formation from acetaldehyde, consistent with the similar amounts of ethanol detected in both cultures (Fig. 2B). Similarly, at/after the diauxic-shift where the cytosolic Adh2 is the major ethanol oxidizer, Adh activities displayed no significant difference (Fig. 3A). On the contrary, during all the growth phases analyzed, Ald activity levels were higher in the mutant compared with the wt (Fig. 3B). In particular, a great increase was observed for Ald6 which is the major cytosolic isoform and is not glucose-repressed 27 compared with that of the mitochondrial counterparts Ald5 and Ald4 (Fig. 3C and D) indicating that the mutant exhibits an increased ability to generate acetate, especially the cytosolic one, which can be used as substrate to produce acetyl-CoA. Accordingly, in the mutant, the nucleocytosolic Acs2-mediated pathway is upregulated during chronological aging 8. Moreover, an increased cytosolic acetate pool can also account for the extracellular acetate detected in the *mpc1*∆ culture (Fig. 2C) whose prolonged accumulation, however, indicates that the flux towards its formation exceeds its utilization. This takes place despite the upregulation of Acs2 enzymatic activity 8 suggesting that the enzyme and/or the flux downstream is/are working at maximum capacity in line with data which show that increase in Acs activity does not result in enhanced acetate utilization 28,29.

### Lack of Mpc1 is accompanied by an increase of malic enzyme activity and a decrease in respiration

**Figure 4:**
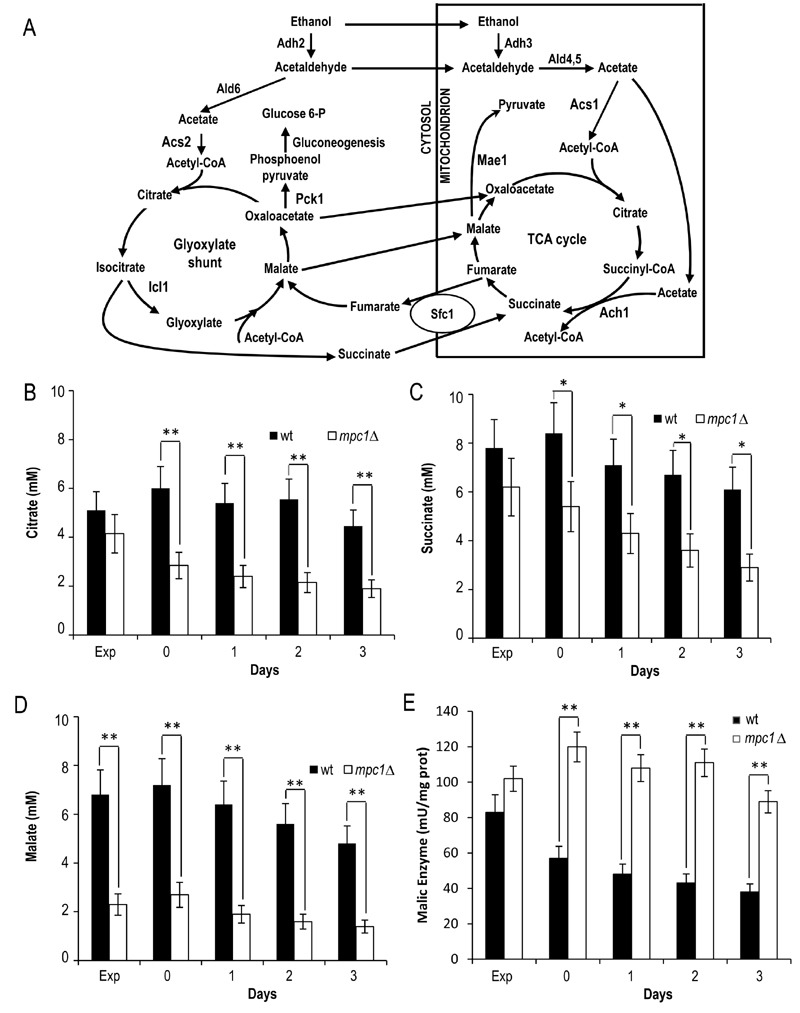
FIGURE 4:Lack of Mpc1 results in low levels of TCA cycle intermediates and enhanced malic enzyme activity. **(A) **Scheme of the TCA cycle and of the glyoxylate shunt. Ach1, acetyl-CoA hydrolase 1; Acs, acetyl-CoA synthase; Adh, alcohol dehydrogenase; Ald, aldehyde dehydrogenase; Icl1, isocitrate lyase 1; Mae1, malic enzyme; Pck1, phosphoenolpyruvate carboxykinase 1; Sfc1, succinate-fumarate carrier. Wt and* mpc1*Δ cells were grown as in Figure 2 and at the indicated time points the concentrations of citrate **(B)**, succinate **(C)** and malate **(D)** were measured. The bar chart of malic enzyme activity **(E)** is also reported. Exp, exponential growth phase. Day 0, diauxic shift. Data refer to mean values determined in three independent experiments. SD is indicated. * P ≤ 0.05 and ** P ≤ 0.01.

Starting from these results, we focused on the mitochondrially localized TCA cycle which can be fed with acetyl-CoA generated either from acetate or following oxidation of mitochondrial pyruvate. We measured the levels of citrate, succinate and malate which are intermediates of this cycle but also metabolic connections with the glyoxylate shunt. This is an anaplerotic device of the TCA cycle which allows the formation of C4 units from C2 units (acetate) by bypassing oxidative decarboxylation (Fig. 4A) 30. At/after the diauxic shift, a clear global decrease was observed for all three intermediates in the *mpc1*∆ cells compared with the wt counterparts (Fig. 4B-D). This decrease was particularly marked for malate which can be used to generate pyruvate in the mitochondria for biosynthetic purposes. This reaction of oxidative decarboxylation is catalyzed by the mitochondrial malic enzyme encoded by *MAE1* 31. As shown in Fig. 4E, in the* mpc1*∆ mutant during the post-diauxic phase, the malic enzyme activity was doubled in comparison with the wt, suggesting that the impairment of pyruvate import into mitochondria linked to Mpc1 loss is compensated by a flux redirection through the Mae1-dependent alternative route. This can explain the severe growth defect observed by 22 when the *mpc1*∆ allele was combined with *MAE1 *deletion.

**Figure 5:**
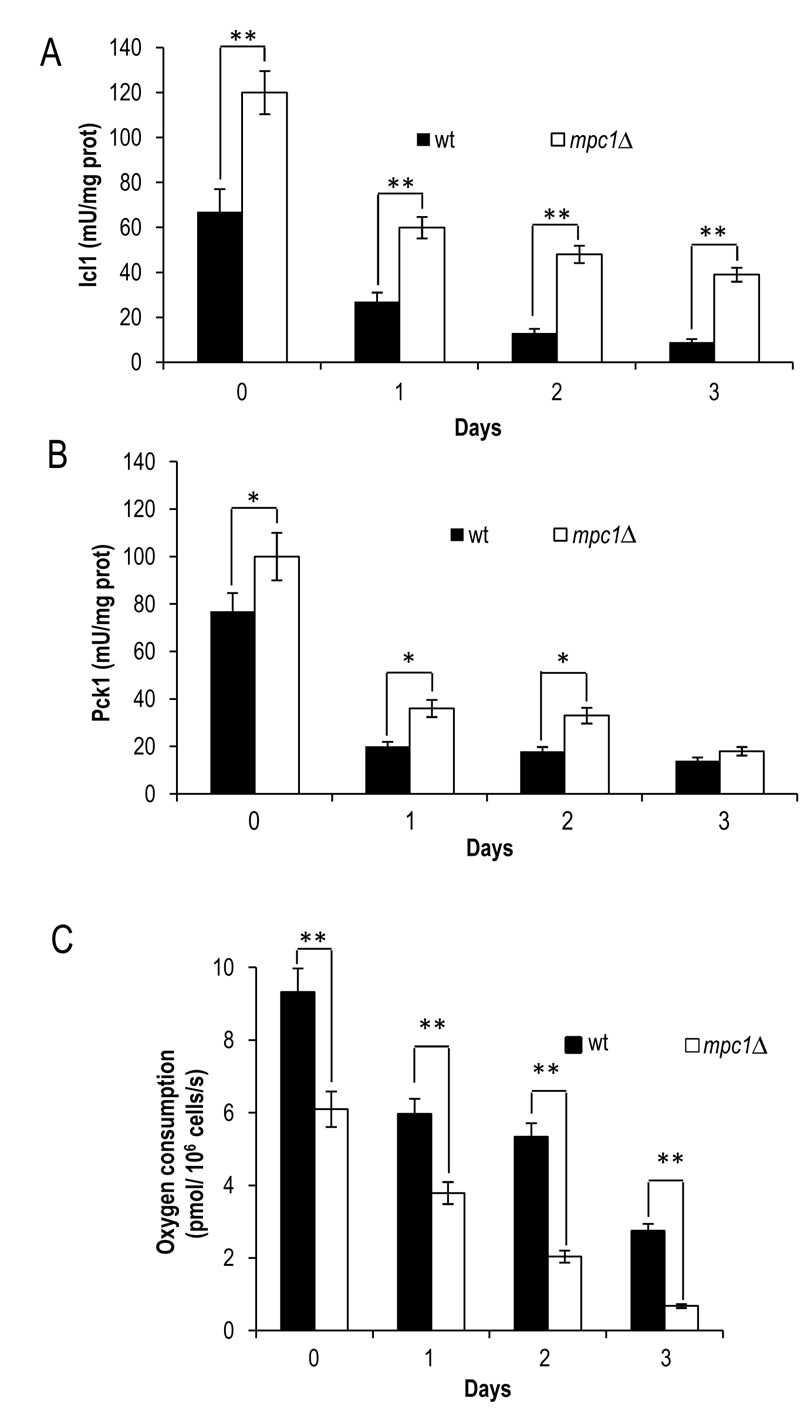
FIGURE 5:*MPC1* inactivation increases glyoxylate/gluconeo-genesis and reduces respiration during chronological aging. At the indicated time points Icl1 **(A)** and Pck1 **(B)** enzymatic activities of wt and *mpc1*Δ mutant cells were measured. In parallel, cellular respiration **(C)** was also monitored. Day 0, diauxic shift. Data refer to mean values determined in three independent experiments. SD is indicated. * P ≤ 0.05 and ** P ≤ 0.01.

Moreover, when cells switched to a respiration-based metabolism by using ethanol and acetate, the glyoxylate shunt becomes operative and begins replenishing the TCA cycle intermediates. In addition, during growth on C2 compounds, this shunt is the exclusive source of oxaloacetate which is the substrate of phosphoenolpyruvate carboxykinase (Pck1), the key enzyme of gluconeogenesis 32. Measurements of the enzymatic activities of isocitrate lyase (Icl1), which is one of the unique enzymes of the glyoxylate shunt, and Pck1 indicated that these activities were higher in *mpc1*∆ cells compared with wt ones (Fig. 5A and B). Concomitantly, in *mpc1*∆ cells cellular respiration decreased (Fig. 5C). Icl1 is localized in the cytosol and from isocitrate it generates succinate and the name-giving metabolite glyoxylate which condenses with acetyl-CoA yielding malate. The last one can return to the mitochondria (Fig. 4A). Similarly, the major fate of cytosolic succinate is assumed to be its transfer into mitochondria 30. Moreover, its transport by the Sfc1 carrier provides cytosolic fumarate for conversion to malate which can be used for gluconeogenesis 33. Thus, taken together, these data indicate that in the *mpc1*∆ mutant, an increase in the glyoxylate shunt might represent an increase in metabolite feeding from the cytosol to support a mitochondrial impaired TCA cycle. In this context, the cytosol of the mutant can provide the metabolic environment required to fulfill the increased requirement of substrates for the glyoxylate shunt. In fact, the end-product of the Acs2 synthetase, which is increased in the mutant 8, is the nucleocytosolic acetyl-CoA. In the cytosol, this metabolite, following condensation with oxaloacetate, produces citrate which is then isomerized to isocitrate (the substrate of Icl1). In addition, the cytosol of the mutant might also be a suitable environment which can “promote” Pck1 enzymatic activity. In fact, Pck1 is acetylated by Esa1 and this acetylation is required for its enzymatic activity: an increase of Pck1 enzymatic activity is associated with an increase of the acetylated form of the enzyme 34,35. Accumulating evidence indicates that the availability of acetyl-CoA, the donor substrate for acetylation, can be a metabolic input for the acetylation itself 36,37,38, so it is reasonable to hypothesize that changes of acetyl-CoA levels may also influence Esa1 activity.

### Carnitine restores chronological longevity of the *mpc1*∆ mutant 

**Figure 6:**
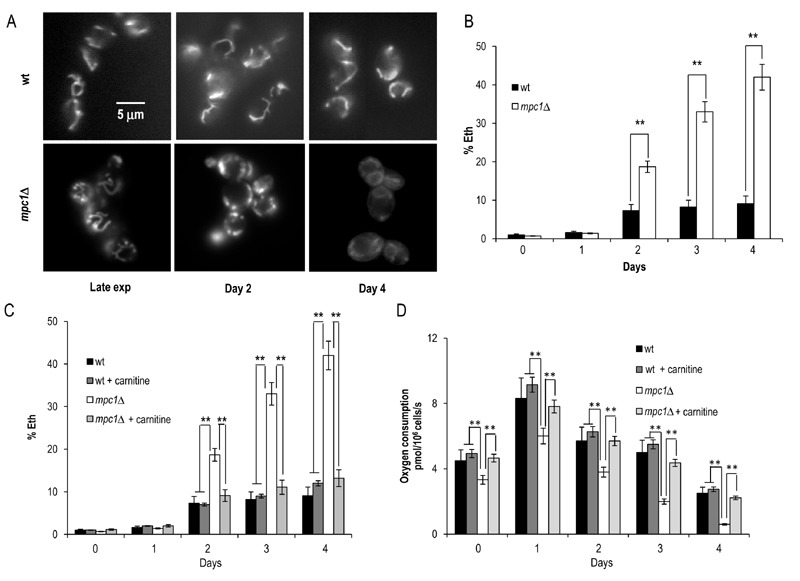
FIGURE 6: Chronologically aging *mpc1*∆ cells display damaged mitochondria. **(A)** Representative images of wt and *mpc1*Δ cultures of Figure 2 stained with DiOC_6_ to visualize mitochondrial membranes. Morphologies of the mitochondria in late exponential phase (Late exp) are also shown. The same cultures were assessed for the presence of intracellular superoxide by conversion of non-fluorescent dihydroethidium into fluorescent ethidium (Eth). Summary graphs of the percentage of fluorescent/superoxide positive cells (% Eth) are reported **(B).** **(C)** Summary graphs of % Eth cells and **(D)** cellular respiration determined in wt and *mpc1*Δ cultures grown in minimal medium/2% glucose supplemented with carnitine (10 mg/L). Day 0, diauxic shift. For the determination of Eth cells, evaluation of about 1000 cells for each sample (three technical replicates) in three independent experiments was performed. SD is indicated. * P ≤ 0.05 and ** P ≤ 0.01.

**Figure 7:**
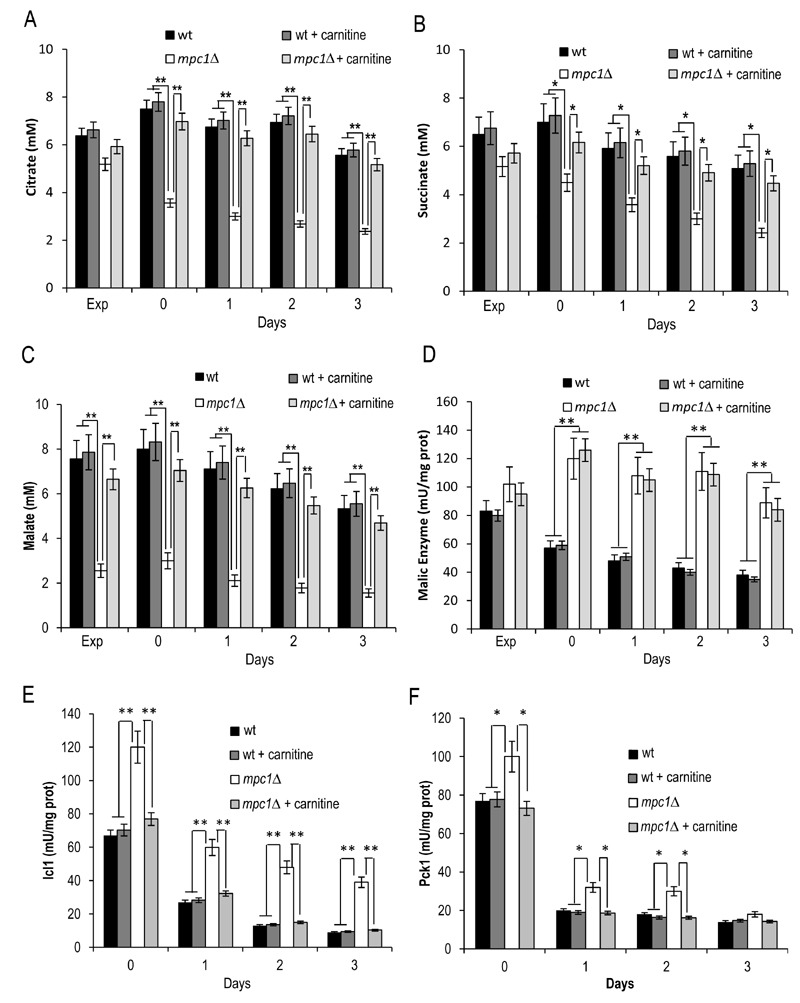
FIGURE 7: Carnitine increases the levels of the TCA cycle intermediates in the *mpc1*∆ mutant. Wt and *mpc1Δ* cells were grown in minimal medium/2% glucose supplemented with carnitine (10 mg/L) and, at the indicated time points, the concentrations of citrate **(A)**, succinate **(B)** and malate **(C)** were measured together with Mae1 **(D)**, Icl1 **(E)** and Pck1 **(F)** enzymatic activities. Day 0, diauxic shift. Data refer to mean values determined in three independent experiments. SD is indicated. * P ≤ 0.05 and ** P ≤ 0.01.

**Figure 8:**
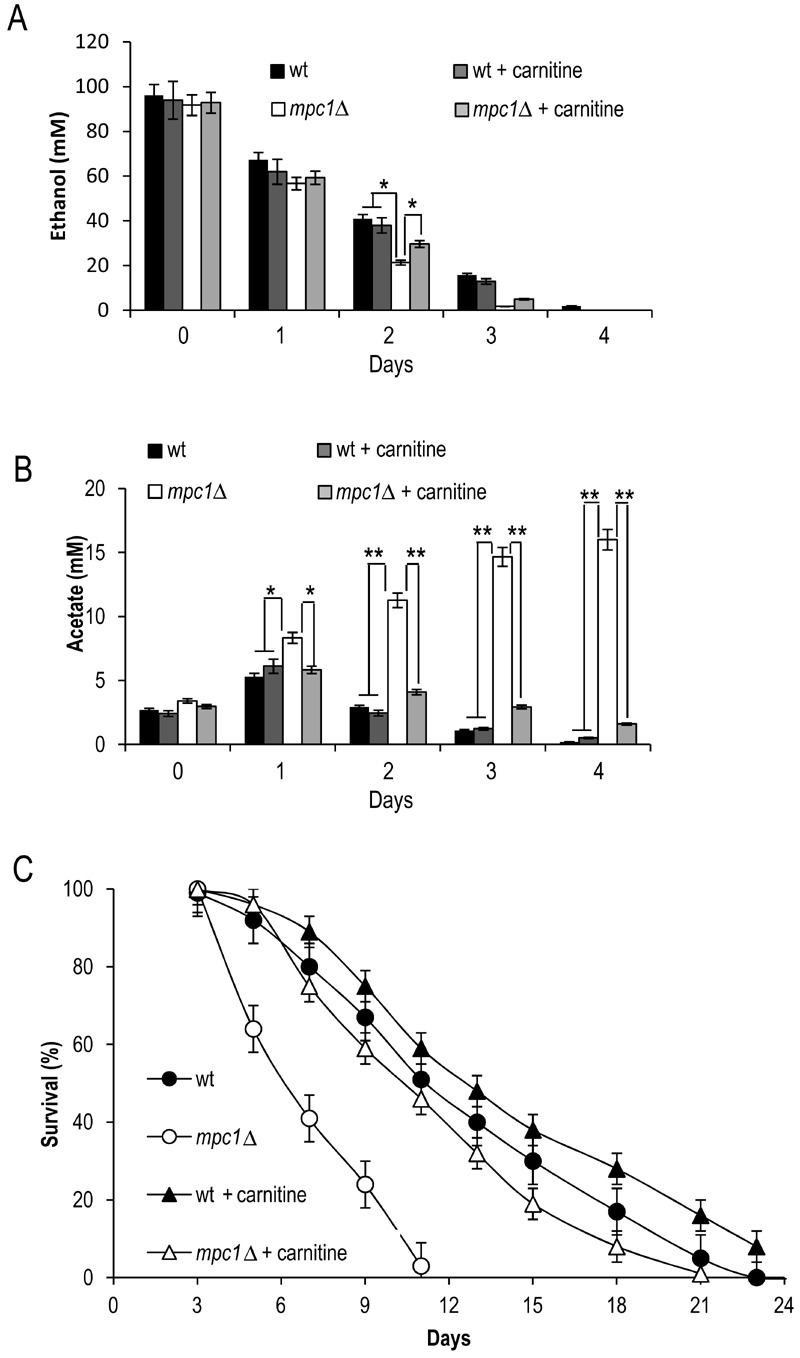
FIGURE 8: Carnitine promotes acetate utilization in the* mpc1*∆ mutant in concert with increased CLS. Bar charts of extracellular ethanol **(A)** and acetate **(B)** concentrations at different time points after the diauxic shift (Day 0) measured for cells grown as in Figure 7. **(C)** CLS of the same cells determined as in Figure 2. Data refer to mean values of three independent experiments. SD is indicated. * P ≤ 0.05 and ** P ≤ 0.01.

After the diauxic shift, a metabolic change from fermentation to respiration takes place implying that energy metabolism relies on mitochondrial functionality. Since in the *mpc1*∆ cells we observed a decrease in respiration, we decided to analyze mitochondrial membrane potential and morphology by using the fluorescent dye, 3,3”-dihexyloxacarbocyanine iodide (DiOC_6_) 39. In fact, mitochondrial morphology reflects the functional status of mitochondria and is regulated by the orchestrated balance of two opposing events: fission and fusion of mitochondria 40. As shown in Fig. 6A, a typical tubular network was observed for wt cells whereas for the mutant fluorescent punctiform structures appeared at Day 2 after the diauxic shift. These structures are indicative of mitochondrial fragmentation and are linked to an elevated activity of the mitochondrial fission machinery 41. In addition to an altered morphology, the mitochondria of the mutant displayed a time-dependent reduction in membrane potential and, at Day 4, did not accumulate DiOC_6_ (Fig.6A). Mitochondrial dysfunctions are intrinsically related to reactive oxygen species (ROS) of which superoxide anion is one of the most potentially harmful. This radical derives mainly from leakage of electrons from the respiratory chain and, among others, can target mitochondria with detrimental effects 42,43. Chronologically aging *mpc1*∆ cells had a higher ROS content, measured as the superoxide-driven conversion of non-fluorescent dihydroethidium (DHE) into fluorescent ethidium (Eth), compared with that of the wt cells (Fig. 6B). Notably, culturing *mpc1*∆ cells in a carnitine-supplemented medium was sufficient to avoid this phenomenon (Fig. 6C). Moreover, oxygen consumption measurements indicated that in these cells cellular respiration increased (Fig. 6D). In *S.cerevisiae*, carnitine is involved in a process referred to as the carnitine shuttle which allows the transport of acetyl-CoA to the mitochondria. This transport system which is non-functional unless carnitine is supplied with the medium 44,45, involves the transfer of the acetyl moiety of acetyl-CoA to carnitine and the subsequent transport of the acetylcarnitine to the mitochondria. Here, a mitochondrial carnitine acetyltransferase catalyses the reverse reaction generating carnitine and acetyl-CoA which enters the TCA cycle 44,46. As shown in Fig. 7A-C, the supplemental carnitine did not significantly affect the levels of citrate, succinate and malate in the wt whilst this was not the case for the *mpc1*∆ mutant where the levels of all three intermediates increased and were restored to wt-like ones. No effect was observed on the malic enzyme activity which at/after the post diauxic shift in the* mpc1*∆ cells was still the double of that of the wt (Fig. 7D) suggesting that the presence of carnitine does not abolish the Mae1-dependent flux towards mitochondrial pyruvate generation. Concomitantly, in the* mpc1*∆ cells the enzymatic activities of Icl1 and Pck1 were reduced to the physiological levels measured in the wt (Fig. 7D and E). Thus, all this suggests that in the *mpc1*∆ mutant the activation of the carnitine shuttle can properly feed the TCA cycle by supplying acetyl-CoA to the mitochondria. Hence, the compensative metabolite feeding from the cytosol provided by the glyoxylate shunt seems to be no longer required. Moreover, following carnitine supplementation, during the post-diauxic phase no effect was observed on the ethanol consumption in both the wt and the mutant strains (Fig. 8A). Similarly, the acetate utilization in the wt was not affected, while in the *mpc1*∆ mutant its utilization was promoted (Fig. 8B). This indicates that in the latter the activation of the carnitine shuttle and the consequent acetyl-CoA transport to the mitochondria can result in an enhancement in the flux downstream from the acetate activation allowing acetate utilization. Finally, in the *mpc1*∆ mutant these metabolic changes matched the almost completely restored chronological longevity (Fig. 8C).

In conclusion, these data collectively indicate that the lack of the Mpc1 transporter brings about a chain of metabolic events which, in order to counteract the decrease of the pyruvate supply in the mitochondria, by influencing the global acetyl-CoA metabolism ultimately restrict cell survival during chronological aging. In particular, after the diauxic shift when cells utilize the earlier produced ethanol/acetate and increase their respiration demand, one of the metabolic traits of the *mpc1*∆ mutant is a TCA cycle operating in a “branched” fashion with a propensity to shunt intermediates towards pyruvate generation via the malic enzyme. This kind of not-complete cyclic functioning of the TCA cycle by depleting it of intermediates influences not only the respiration, which is reduced in the mutant, but also might reduce mitochondrial acetyl-CoA pool. In fact, a TCA cycle characterized by low levels of intermediates (Fig. 4B-D) generates less succinyl-CoA. This is the substrate for the CoA-transferase's reaction from succinyl-CoA to acetate, catalyzed by Ach1 in cells released from glucose repression 20. In the mitochondria, this reaction allows the production of acetyl-CoA 8. Moreover, the CoA-transferase's reaction is using acetate as acceptor, which implies that Ach1 is also important for acetate detoxification and mitochondrial functionality during chronological aging 47. Consequently, in a condition where acetate-generating activities of Ald4/Ald5 are increased, as it is the case in the mutant, a reduction in CoA-transferase's enzymatic activity could play a causative role in promoting/enhancing the mitochondrial damage observed in the mutant.

Furthermore, since during the utilization of ethanol and acetate, the sole possible route for the net synthesis of C4 dicarboxylic acids for replenishing the TCA cycle of intermediates is the glyoxylate shunt, it follows that in the *mpc1*∆ cells this anaplerotic shunt is enhanced in order to keep the “branched” TCA cycle functioning. In turn, it follows that the pathway providing the cytosolic acetyl-CoA must be increased to support an enhanced glyoxylate demand. In line with this, the cytosolic Ald6 enzymatic activity is increased (Fig. 3C) and hyperactivation of the Acs2 activity has been detected 8. Interestingly, this synthetase is responsible not only for supplying acetyl-CoA for carbon metabolism, but also for protein acetylation, particularly of histones 19. In addition, it has been shown that during chronological aging, upregulation of Acs2 activity culminates in histone H3 hyperacetylation associated with transcriptional downregulation of several autophagy-essential *ATG* genes 48. In this context, *mpc1*∆ cells display an age-dependent loss of autophagy 8; this feature, given the reciprocal cross-talk between autophagy and mitochondria, can negatively affect the removal of the damaged mitochondria of the mutant and consequently contribute to its inability to maintain proper cellular homeostasis during the aging process. Notably, Ald6, which is responsible of generating cytosolic acetate, is degraded preferentially by autophagy 49 and the persistence of its enzymatic activity seems to be disadvantageous for the survival during nitrogen starvation 50. Thus, *mpc1*∆ cells make up for their impairment in mitochondrial pyruvate with a metabolic rewiring in which the pro-aging outcome prevails.

## MATERIALS AND METHODS

### Yeast strains and growth conditions

The *mcp1*∆ mutant (*mcp1*∆::*HIS3*) was generated by PCR-based methods in a BY4741 background (*MAT*a* his3*∆*-1 leu2*∆*-0 met15*∆*-0 ura3*∆*-0) *and the accuracy of gene replacement was verified by PCR with flanking and internal primers. At least two different clones were tested for any experiment. Yeast cells were grown in batches at 30°C in minimal medium (Difco Yeast Nitrogen Base without amino acids, 6.7 g/L) with 2% glucose and the required supplements added in excess to a final concentration of 200 mg/L, except for leucine at 500 mg/L to avoid auxotrophy starvation 51,52. L-carnitine (Sigma) was supplemented to a concentration of 10 mg/L. Strains were inoculated at the same cellular density (culture volume no more than 20% of the flask volume) and growth was monitored by determining cell number using a Coulter Counter-Particle Count and Size Analyser, as described 53. Duplication times (Td) were obtained by linear regression of the cell number increase over time on a semi-logarithmic plot.

### CLS determination

Survival experiments in expired medium were performed on cells grown in minimal medium/2% glucose and the required supplements as described above. During growth, cell number and extracellular glucose, ethanol and acetic acid were measured in order to define the growth profile (exponential phase, diauxic shift, post-diauxic phase and stationary phase) of the culture (Fig. S1). Cell survival was monitored by harvesting aliquots of cells starting with 72 h (Day 3, first age-point) after the diauxic shift (Day 0). CLS was measured according to 51 by counting colony-forming units (CFUs) every 2-3 days. The number of CFUs on Day 3 was considered the initial survival (100%).

### Metabolite measurements and enzymatic assays 

At designated time points, aliquots of the yeast cultures were centrifuged and both pellets (washed twice) and supernatants were frozen at −80°C until used. Rapid sampling for intracellular metabolite measurements was performed according to the leakage-free cold methanol quenching method developed by 54 in which pure methanol at ≤ -40°C and a ratio of cell culture to quenching solvent of 1:5 (final methanol concentration ≥ 83%) were used. Metabolites from the cell pellets were extracted in 5 ml of a solution of 75% (v/v) boiling absolute ethanol containing 0.25 M Hepes, pH 7.5, as described in 55. The concentrations of glucose, ethanol, acetate, pyruvate, citrate, succinate and malate were determined using enzymatic assays (K-HKGLU, K-ETOH, K-ACET, K-PYRUV, K-SUCC, K-CITR and K-LMALR kits from Megazyme). Ethanol specific consumption rate (qEtOH), expressed in mmol•g•DW^ -1^•h^-1^, was calculated from measured cell dry weights (DWs) and extracellular ethanol concentrations. DW was measured as described 56.

All the enzymatic activities were assayed immediately after preparation of cell-free extracts. Cells were resuspended in 100 mM potassium phosphate buffer, pH 7.5, containing 2 mM MgCl_2_ and 1 mM dithiothreitol and broken with acid-washed glass beads by shaking on a vortex for several cycles interspersed with cooling on ice. The activities of cytosolic and mitochondrial aldehyde dehydrogenase (Ald) were measured as described by 57, of alcohol dehydrogenase (Adh) according to 58, of phosphoenolpyruvate carboxykinase (Pck1) and isocitrate lyase (Icl1) as in 34. Malic enzyme activities were determined according to 31 with either 0.4 mM NAD^+^ or NADP^+^ as the redox cofactor. The enzymatic activity was measured in the decarboxylation direction to avoid interference with pyruvate decarboxylase and Adh. Total protein concentration was estimated using the BCA^TM^ Protein Assay Kit (Pierce).

### Oxygen consumption and fluorescence microscopy

The basal oxygen consumption of intact cells was measured at 30°C using a ‘Clark-type’ oxygen electrode in a thermostatically controlled chamber (Oxygraph System, Hansatech Instruments, Norfolk, UK) as previously reported 25. Data were recorded at sampling intervals of 1 s (Oxygraph Plus software, Hansatech Instruments, Norfolk, UK). All assays were conducted in biological triplicate.

ROS were detected with dihydroethidium (DHE, Sigma) according to 59. The mitochondrial membrane potential was assessed by staining with DiOC_6 _(Molecular Probes, Invitrogen), according to 39; cells were also counterstained with propidium iodide to discriminate between live and dead cells. A Nikon Eclipse E600 fluorescence microscope equipped with a Leica DC 350F ccd camera was used. Digital images were acquired using FW4000 software (Leica).

### Statistical analysis of data

All values are presented as the mean of three independent experiments with the corresponding Standard Deviation (SD). Three technical replicates were analyzed in each independent experiments. Statistical significance was assessed by one-way ANOVA test. P value of ≤ 0.05 was considered statistically significant.

### SUPPLEMENTAL MATERIAL

Click here for supplemental data file.

All supplemental data for this article are also available online at http://microbialcell.com/researcharticles/rewiring-yeast-acetate-metabolism-through-mpc1-loss-of-function-leads-to-mitochondrial-damage-and-decreases-chronological-lifespan/.
