# Supplementary material for: Rewiring yeast acetate metabolism through MPC1 loss of function leads to mitochondrial damage and decreases chronological lifespan
Source: Microb Cell. 2014 Nov 18;1(12):393–405. doi: 10.15698/mic2014.12.178 (PMC5349135; doi:10.15698/mic2014.12.178)
Supplement: Supplementary file 1 [file mic-01-393-s01.pdf]

SUPPLEMENTARY

FIGURE S1: Wild type (wt) and *mpc1Δ* mutant cells were grown in minimal medium/2% glucose and the required supplements in excess (see Materials and Methods). Cell growth was monitored by counting cell number over time and, in parallel, the extracellular concentration of glucose and ethanol were measured in medium samples collected at different time-points in order to define the growth profile (exponential phase, diauxic shift, post-diauxic phase and stationary phase) of the cultures. One representative experiment is shown.

FIGURE S2: The wt BY4741 strain (*his3Δ-1 leu2Δ-0 met15Δ-0 ura3Δ-0)* and its derivative *mpc1Δ* mutant (*mcp1Δ*::*HIS3*) were grown in minimal medium/2% glucose containing the same supplements (His, Leu, Met and Ura) in excess and followed up to stationary phase. **(A)** CLS of wt and *mpc1Δ* mutant cells. At each time-point, survival was determined by colony-forming capacity. 72 h after the diauxic shift (Day 3) was considered the first age-point. Day 0, diauxic shift. Data refer to mean values of three independent experiments. Standard deviations (SD) are indicated. Bar charts of extracellular ethanol **(B)** and acetate **(C)** concentrations at different time-points after the diauxic shift (Day 0). In parallel, intracellular **(D)** and extracellular **(E)** pyruvate concentrations were measured. Exp, exponential growth phase. Data refer to mean values of three independent experiments. SD is indicated. Statistical significance, assessed by one-way ANOVA test, is indicated (* $P \leq 0.05$ and ** $P \leq 0.01$).

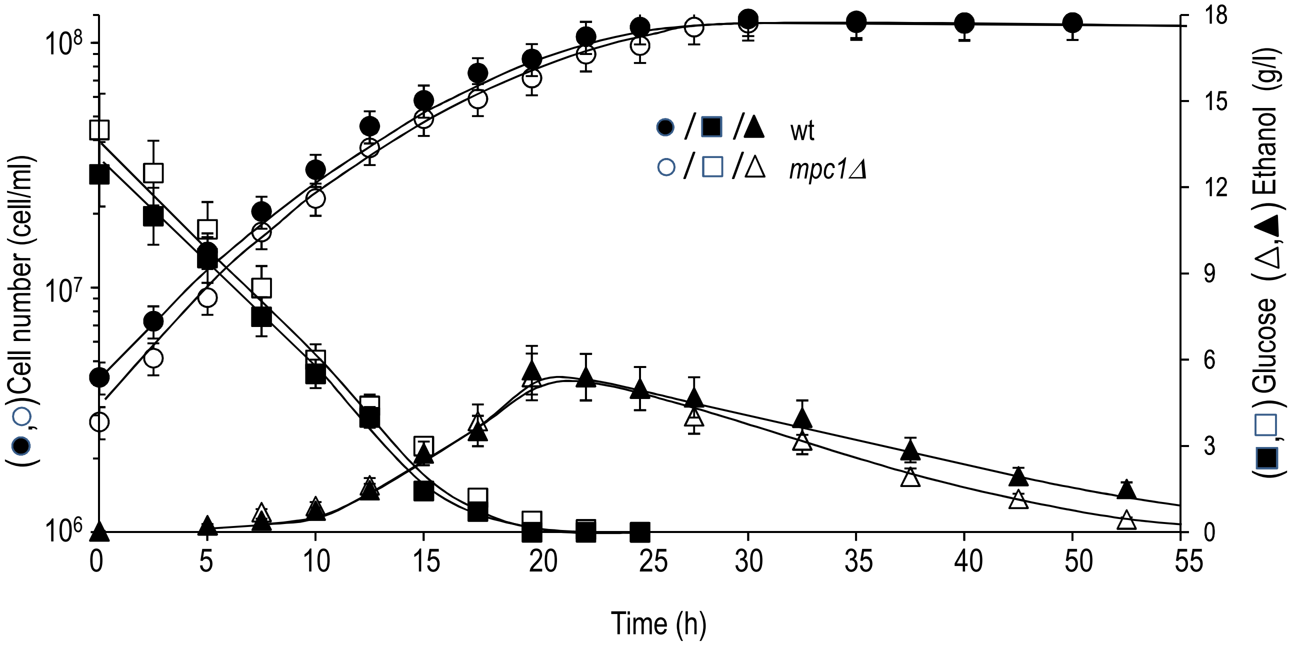

Fig. S1

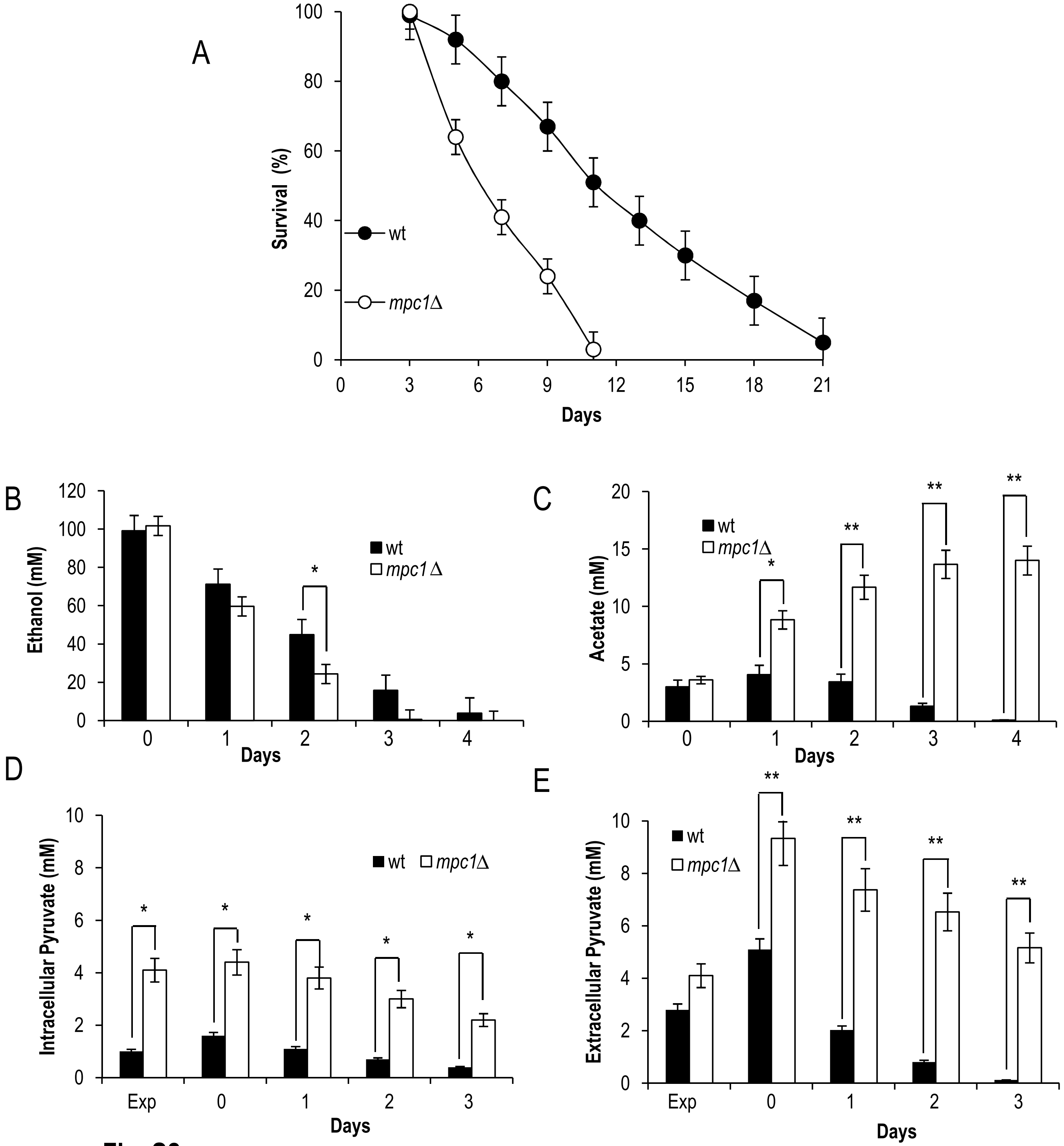
A
Survival (%)
100
80
60
40
20
0
0
3
6
9
12
15
18
21
Days
wt
mpc1Δ
B
Ethanol (mM)
120
100
80
60
40
20
0
1
2
3
4
Days
wt
mpc1Δ
*
C
Acetate (mM)
20
15
10
5
wt
mpc1Δ
*
**
D
Intracellular Pyruvate (mM)
10
8
6
4
2
Exp
wt
mpc1Δ
E
Extracellular Pyruvate (mM)

Fig. S2
